# Optimizing internal control in public hospital supply chain: a game theory-based approach

**DOI:** 10.3389/fpubh.2023.1240757

**Published:** 2023-10-18

**Authors:** Zhihao Yu

**Affiliations:** College of Engineering, Computing and Cybernetics, Australian National University, Canberra, ACT, Australia

**Keywords:** internal control, supply chain, combination weighting method of game theory, entropy weighting method, analytic hierarchy process

## Abstract

**Objective:**

Our study aims to enhance the precision of internal control construction within public hospital supply chains and minimize the subjective bias influence. We have integrated the game theory combination weighting method into the design of internal control paths and based on this, developed a series of pioneering solutions. This innovative approach is anticipated to heighten the effectiveness and scientific rigor of the internal control design scheme within the supply chain.

**Method:**

Firstly, we utilized literature review and expert interviews to delve into the key factors of public hospital supply chain internal control, forming an index system for public hospital supply chain internal control that aligns with current informatization requirements. Subsequently, we incorporated the Game Theory Combination Weighting Method into this study. By means of the Analytic Hierarchy Process and the Entropy Weighting Method we determined the subjective and objective weights of each index and obtained their comprehensive weights through the Game Theory Combination Weighting Method. Then, based on the analysis results, we designed a series of internal control construction schemes and implemented these schemes at Weifang Maternal and Child Health Hospital between 2019 and 2023. Finally, using the Fuzzy Comprehensive Evaluation Method to assess and compare the actual effects before and after the implementation of the schemes, thereby validating the effectiveness of the Game Theory Combination Weighting Method in the design of the internal control path of public hospital supply chains.

**Results:**

The fuzzy comprehensive evaluation results for the years 2019 and 2023 demonstrated that after implementing our design schemes using the Game Theory Combination Weighting Method, the hospital’s satisfaction in aspects such as plan-side control, purchase-side control, asset-side control, expenditure business control, and contract management control has significantly improved.

**Conclusion:**

Our research indicates that the Game Theory Combination Weighting Method is applicable to the path design of internal control links in public hospital supply chains. This method has effectively enhanced the targeted improvement of weak links within the construction of internal controls in the supply chain of public hospitals and is of great significance for improving the scientific nature of supply chain internal control management.

## 1. Introduction

The more widely used definition of internal control is proposed by the National Anti-fraudulent Financial Reporting Committee (Committee of Sponsoring Organizations of the Treadway Commission, COSO Committee) in “Internal Control: An Integrated Framework” (1), that is, internal control is the process of providing reasonable assurance for the achievement of objectives such as operating results, authenticity of financial reports and compliance with applicable laws. The supply chain is an integrated manufacturing process wherein raw materials are converted into final products, then delivered to customers (2, 3). The effectiveness and efficiency of the supply chain are largely contingent upon its internal control mechanisms. Internal controls not only ensure the smooth operation of each link in the supply chain but also offer risk management and decision-making support for supply chain management, thereby guaranteeing the quality and safety of medical services (4).

Given the distinct characteristics of the healthcare sector, hospital supply chains differ significantly from traditional supply chains in other industries. From a quality perspective, hospital supply chains emphasize maintaining high standards due to its direct bearing on patient safety and well-being. Any deviation in quality can lead to severe health risks. From a performance perspective, hospital supply chains prioritize timely delivery, inventory management, and the prevention of medication and medical equipment shortages. In contrast, traditional supply chains often prioritize cost-effectiveness and meeting market demands. These significant differences make hospital supply chains more in need of avoiding the generation of subjective biases compared to traditional supply chains. Such biases can impose significant financial strains on hospitals and carry profound, irreversible consequences for the quality and safety of medical services. For instance, selecting an inappropriate drug can compromise therapeutic outcomes or lead to severe side effects; opting for unsuitable medical equipment can increase surgical risks; and delays in the supply chain can cause shortages of crucial drugs or equipment, impacting urgent surgeries or treatments. These issues not only threaten patient health and safety but also risk damaging the hospital’s reputation and may result in legal disputes. Regrettably, the internal control within public hospitals currently predominantly relies on the subjective decisions of managers, and the control schemes are usually determined by discussions among multiple individuals involved in its administrative management (5). This may result in deviations between certain decisions and actual needs, consequently impacting the operational efficiency of hospitals. Despite these issues, effective solutions are lacking. Therefore, the urgent task in public hospital supply chain management is to design and develop a method that can precisely and objectively evaluate and optimize the internal control of public hospital supply chains.

In recent years, a plethora of studies have been dedicated to exploring how to reduce subjective biases in hospital internal controls. Patil and Kant (6) propose a framework based on fuzzy analytical hierarchy process (AHP) and fuzzy technique for order performance by similarity to ideal solution (TOPSIS) to identify and rank the solutions of knowledge management adoption in supply chain and overcome its barriers; Samvedi et al. (7) proposed to use the analytical hierarchy process (AHP) and the fuzzy technique for order preference by similarity to the ideal solution (TOPSIS) to assess the risk in the supply chain. Venkatesh et al. (8) proposed to use the AHP-TOPSIS method to evaluate the supplier selection module in the humanitarian aid supply chain, thereby reducing the impact of subjective biases; Kumar and Singh (9) delved into the application of an integrated approach combining fuzzy Analytical Hierarchy Process (fuzzy AHP) and TOPSIS to evaluate the performance of global third-party logistics service providers for enhancing supply chain management effectiveness.

Previous studies primarily utilized either individual quantitative analysis or combined the Analytic Hierarchy Process (AHP) with the Technique for Order of Preference by Similarity to Ideal Solution (TOPSIS) to assess internal controls within supply chains, aiming to mitigate subjective biases. While TOPSIS can mitigate the issue of subjective bias to some extent and provide a structured decision-making framework, the reliance of this method on human judgment when determining criterion weights and evaluating alternative solutions may still introduce biases. These biases, stemming from personal perceptions, experiences, or cognitive inclinations, can jeopardize the assessment’s reliability and validity, particularly when evaluators’ judgments diverge. Recognizing these challenges, we aimed to bolster the evaluation’s robustness. We incorporated the Game Theory Combination Weighting Method into our study’s hospital internal control assessment of supply chains to counteract the inherent subjective biases of AHP. This method adeptly marries the strengths of AHP with the Entropy Weighting Method’s objectivity, facilitating a more logical computation of indicator weights. It aligns decision-making subjectivity with each indicator’s intrinsic value, ensuring the assessment remains uninfluenced by individual biases, thus enhancing its objectivity, precision, and depth, and producing more steadfast and credible results.

Based on the AHP-EWM (Analytic Hierarchy Process-Entropy Weighting Method) combined predictive model established by Chengguang et al. (10), we first embarked on a comprehensive acquisition and integration of internal control indexes within the public hospital supply chain. This was achieved through extensive literature analysis, questionnaire surveys, and expert interviews, thereby constructing a comprehensive internal control index system for public hospital supply chains. Then, we employed the Analytic Hierarchy Process and Entropy Weighting Method to derive both subjective and objective weights for each index. Subsequently, the Game Theory Combination Weighting Method was adopted to calculate the comprehensive weight of the indexes. Guided by the results of these weight computations, we innovatively proposed a series of internal control schemes for supply chains. These schemes were subsequently put into practice over a four-year period at the Weifang Maternal and Child Health Hospital. Following this implementation, we utilized the Fuzzy Comprehensive Evaluation Method to independently evaluate the hospital’s internal control status within its supply chain, both before and after the introduction of our proposed design scheme. The Evaluation enabled us to test the reliability of the indicator system we established and to verify the feasibility of applying the Game Theory Combination Weighting Method to internal control within public hospital supply chains.

## 2. Method

### 2.1. Hierarchical analysis method

The Analytic Hierarchy Process (AHP) is a structured technique for complex decision-making, widely applied in areas like management, production, traffic safety, and user experience. It decomposes decision-making objectives hierarchically, aiming to quantify and compare multiple projects and indices. The process involves:

1. Constructing a judgment matrix based on relative importance, as shown in Table 1 (11, 12).
2. Using binary comparison to assess related internal control indices at the same level. Managers score the index ratio based on a scale in Table 2, ranging from equal to extreme importance.
3. Engaging experts to compare and score indices at various levels, determining relative importance between criterion and sub-criterion layers, and deriving the judgment matrix. The eigenvector method is applied to the matrix, yielding the weight vector ω. The largest eigenvalue’s eigenvector is normalized to determine each index’s weight (13).

$$\;A\omega =\lambda_{\max}\omega$$

**Table 1:** Construction method of judgment matrix.

*A*	*B_1_*	*B_2_*	*…*	*B_n_*
*B_1_*	*B_11_*	*B_12_*	*…*	*B_1n_*
*B_2_*	*B_21_*	*B_22_*	*…*	*B_2n_*
*…*	*…*	*…*		*…*
*B_n_*	*B_n1_*	*B_n2_*	*…*	*B_nn_*

**Table 2:** Scaling of judgment matrix.

Scale value	Importance	Meaning
1	Equally important	Both indexes are equally important
3	Slightly important	The former metric is slightly more important than the latter
5	Obviously important	The former metric is significantly more important than the latter
7	Strongly important	The former metric is strongly more important than the latter
9	Extremely important	The former metric is definitely more important than the latter
2, 4, 6, 8	Median	The median of the above adjacent judgments
1, 1/2,...,1/9	Reciprocal	To the same extent, the importance of the latter compared with the former

To account for evaluators’ subjective biases, a consistency test is performed on the judgment matrix. This ensures reliable, consistent results and minimizes weight calculation uncertainty (14–16). The formulas used include:


$$\begin{array}{c} \;M_{CI}=\frac{\lambda_{\max}-n}{n-1} \end{array}$$

$$\begin{array}{c} \;M_{CR}=\frac{M_{CI}}{M_{RI}} \end{array}$$

Where *λ_max_* is the maximum eigenvalue and *n* is the matrix order. If *M_CR_ > 0.1*, the matrix’s weight distribution is deemed consistent; otherwise, it’s contradictory.

### 2.2. Entropy weighting method

The entropy weighting method is a decision-making method that determines the weight of each index by comprehensively considering the amount of information provided by the measurement value of each index. This method takes into account the amount of information provided by each factor and calculates the information entropy of each index to measure its contribution to the overall system. The entropy weighting method is often employed as an approach for obtaining the objective weight, which involves determining the weight of each index based on the differences between the values of multiple indexes in the scheme. The specific steps are as follows:

Invite *m* experts to evaluate the *n* indexes, and the evaluation score ranges from 1 to 10. The higher the score, the more important the index is.

Build an evaluation matrix *B*:


$$\begin{array}{c} \;B={\left(b_{ij}\right)}_{m\times n}=\left[\begin{array}{ccc} b_{11} & \cdots & b_{1n}\\ \vdots & & \vdots \\ b_{m1} & \cdots & b_{mn} \end{array}\right] \end{array}$$

Normalize a matrix:


$$\begin{array}{c} \;x_{ij}^{\prime }=\frac{x_{ij}}{\sum_{i=1}^{m}x_{ij}} \end{array}$$

Information entropy value of each index:


$$\begin{array}{c} \;e_{j}=-\frac{1}{\ln m}\overset{m}{\underset{i=1}{\sum }}x_{ij}^{\prime }\ln x_{ij}^{\prime } \end{array}$$

The information utility value of each index:


$$\begin{array}{c} \;d_{j}=1-e_{j} \end{array}$$

Get the objective weight of each index:


$$\begin{array}{c} \;W_{j}=\frac{d_{j}}{\sum_{j=1}^{n}d_{j}} \end{array}$$

### 2.3. Combination weighting method of game theory

Game theory, a branch of operations research, is concerned with the study of decision-making and strategic interactions among agents with conflicting interests. It is a mathematical framework that analyzes the incentives, strategies, and outcomes of situations where multiple participants make decisions. Game theory considers differences between expected and actual behaviors in weight calculations and seeks to identify optimal strategies for achieving desired outcomes. This approach ensures that the overall assessment remains unaffected by the subjective biases of individual evaluators, effectively addressing the inherent subjectivity pitfalls associated with the Analytic Hierarchy Process. The following is the method flow.

First, establish the basic weight vector set Wq = {ω1, ω2,..., ωn} (*q* = 1, 2,..., *p*). Among them, ω is the set of weights determined by the *p* weighting method, *n* is the number of indexes, and *p* is the number of methods for obtaining weights. The comprehensive weight is calculated by the subjective weight obtained by the AHP method and the objective weight obtained by the entropy weighting method, so *p = 2*. Set *α = {α1,α2}* is the linear combination coefficient, then the linear combination of the subjective weight and the objective weight vector is:


$$\begin{array}{c} \;W=\alpha_{1}\omega_{1}{}^{T}+\alpha_{2}\omega_{2}{}^{T} \end{array}$$

Based on the idea of the game aggregation model, two linear combination coefficients are optimized to minimize dispersion to obtain the most satisfactory weight in *W*. The established objective function is:


$$\;\min||\overset{n}{\underset{p=1}{\sum }}\alpha_{p}\omega_{p}{}^{T}-\omega_{p}||$$

Transform the above equation into a system of linear equations that optimize the first derivative condition:


$$\begin{array}{c} \;\left[\begin{array}{cc} \omega_{1}\omega_{1}{}^{T} & \omega_{1}\omega_{2}{}^{T}\\ \omega_{2}\omega_{1}{}^{T} & \omega_{2}\omega_{2}{}^{T} \end{array}\right]\left[\begin{array}{c} \alpha_{1}\\ \alpha_{2} \end{array}\right]=\left[\begin{array}{c} \omega_{1}\omega_{1}{}^{T}\\ \omega_{2}\omega_{2}{}^{T} \end{array}\right] \end{array}$$

Normalize processing *α_1_、α_2_*:


$$\begin{array}{c} \;\{\begin{array}{c} \alpha_{1}{}^{*}=\frac{\alpha_{1}}{\alpha_{1}+\alpha_{2}}\\ \alpha_{2}{}^{*}=\frac{\alpha_{2}}{\alpha_{1}+\alpha_{2}} \end{array} \end{array}$$

Calculate comprehensive weight:


$$\begin{array}{c} \;W=\alpha_{1}{}^{*}\omega_{1}{}^{T}+\alpha_{2}{}^{*}\omega_{2}{}^{T} \end{array}$$

### 2.4. Fuzzy comprehensive evaluation

The fuzzy comprehensive evaluation method is a multi-objective assessment technique rooted in fuzzy mathematics’ membership degree theory. Often termed as fuzzy multi-objective result evaluation, it’s been extensively applied in domains like education, management, medicine, and risk assessment. This method, comprising three stages–setting the scoring criteria, forming the fuzzy rating matrix, and defining the weight vector–effectively manages fuzzy uncertainties, bolstering evaluation accuracy and impartiality. The evaluation process involves determining the comment set V for the plan, assigning its value, establishing the comprehensive weight vector for index levels (17), and constructing the evaluation matrix for relevant sub-criteria. Experts are then engaged to score the target, with the membership degree of comments determined based on their frequency. The matrix is as follows:


$$\begin{array}{c} \;N_{m}=\left(\begin{array}{ccc} X_{11} & \cdots & X_{1y}\\ \vdots & & \vdots \\ X_{x1} & \cdots & X_{xy} \end{array}\right) \end{array}$$

In the formula, *m* is the level of the weight vector, *X* is the membership degree of different comments, *x* is the number of indexes included in the m level, and *y* is the number of subsets of the comment set.

According to the evaluation results of the sub-criteria layer, the weight vector of the criterion layer to the design scheme is calculated, and *m* is the level of the weight vector:


$$\begin{array}{c} \;n_{m}=W_{m}N_{m} \end{array}$$

Construct the criterion-level evaluation matrix:


$$\begin{array}{c} \;n=\left(\begin{array}{c} n_{1}\\ \vdots \\ n_{m} \end{array}\right)=\left(\begin{array}{ccc} W_{1}N_{1} & \cdots & W_{m}N_{1}\\ \vdots & & \vdots \\ W_{1}N_{m} & \cdots & W_{m}N_{m} \end{array}\right) \end{array}$$

Calculate the comprehensive evaluation weight vector:


$$\begin{array}{c} \;H=W_{m}n \end{array}$$

Based on this, the total score of the relevant construction evaluation is obtained:


$$\begin{array}{c} \;P=HV \end{array}$$

## 3. Case study

**Figure 1:**
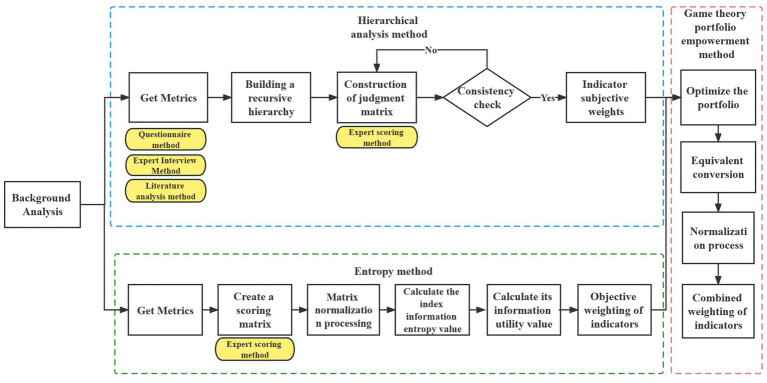
The process of constructing the internal control index system of the supply chain based on combination weighting method of game theory.

In the process of evaluating internal control indexes, evaluators often have personal subjective judgments. To improve the objectivity and reduce uncertainty in the evaluation of internal control indexes, this study listed 12 specific contents related to the internal control business level of public hospitals based on the “Administrative Measures for Internal Control of Public Hospitals” issued by the National Health Commission of China in December 2020 (18). These contents were combined with the businesses included in the hospital supply chain, and internal control indexes were obtained through questionnaires, expert interviews, and literature analysis. Using the game theory combination weighting method, the weights of the internal control indexes of the public hospital supply chain were constructed. The research process is presented in Figure 1.

### 3.1. Index system construction

**Figure 2:**
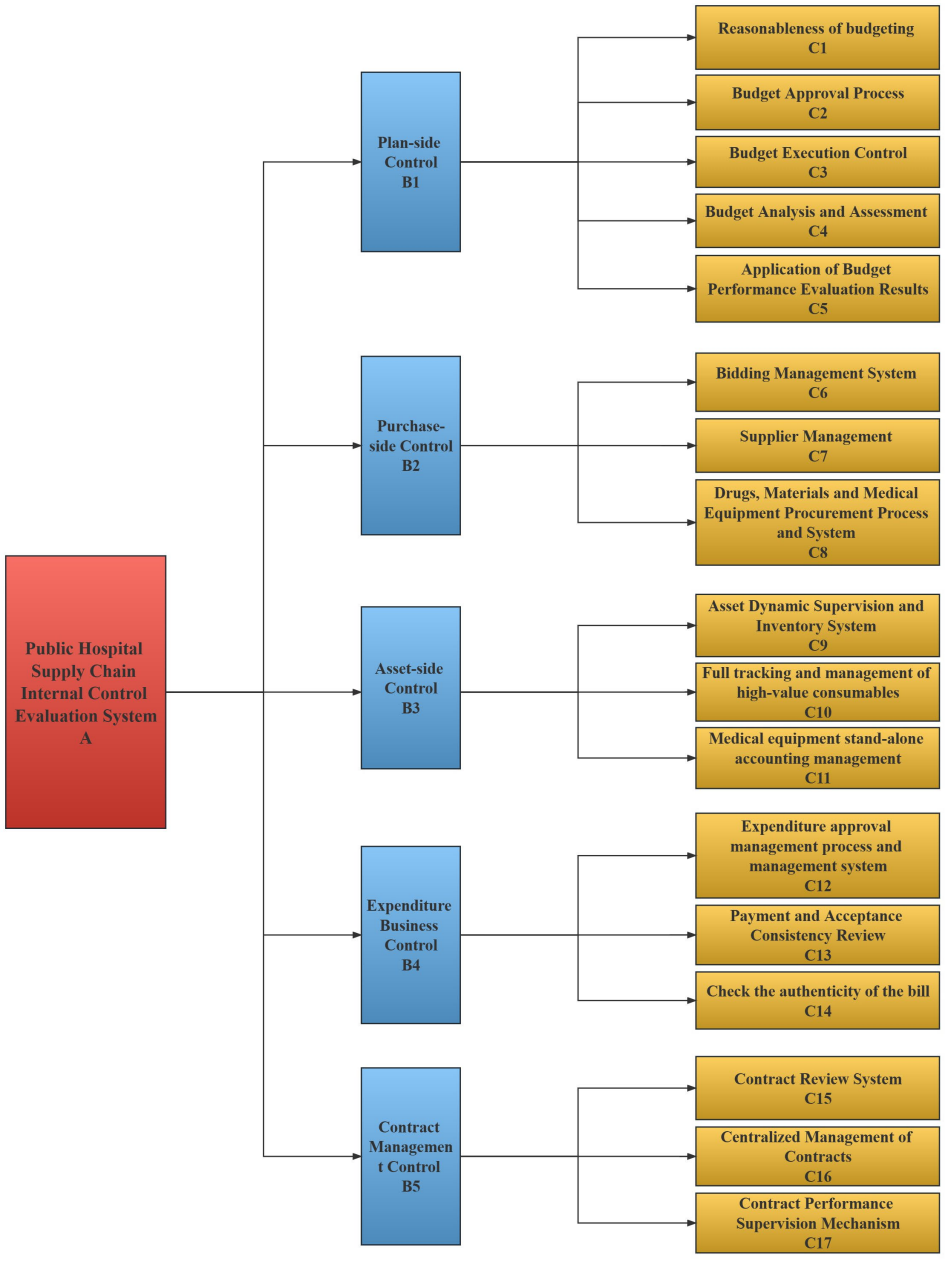
Internal control index system of public hospital supply chain.

Given the intricate nature of the internal control of public hospital supply chains, it is essential to establish a comprehensive and systematic set of indexes. To this end, this study reviewed pertinent literature on supply chains and internal control, consulted with internal control experts, and focused on the structure and processes of public hospital supply chains to identify the necessary internal control measures. To comprehensively analyze and summarize each link, the standard layer was divided into five levels, namely planning, purchasing, asset, expenditure business, and contract management. Through literature analysis, questionnaire survey, and expert interviews, the internal control indexes of the supply chain were obtained. Twenty six indexes were obtained through literature analysis (19, 20); 17 indexes were obtained through questionnaire surveys, and 19 indexes were obtained through expert interviews, for a total of 62 indexes. The indexes obtained through the three methods have a certain degree of repetition. To enhance the coherence and consistency of the index system, this study combined indexes with similar or identical meanings. For instance, “budget execution variance rate” and “budget analysis and assessment” were grouped under the inclusive relationship and referred to collectively as the index of “Budget Analysis and Assessment.” The other indexes were merged in a similar way, and ultimately, a set of 17 indexes were obtained, constituting the internal control index system of the public hospital supply chain, as illustrated in Figure 2. To facilitate comparison, a brief description of each index is presented in Table 3.

**Table 3:** Summary of indexes.

First-level index	Second-level Index	Index description
Plan-side control	Reasonableness of budgeting	The compilation method is scientific, and the content is comprehensive, which is in line with the actual development of the hospital
	Budget Approval Process	Rigorous approval procedure, perfect process, approval step by step, separation of incompatible positions, efficient and in line with internal control requirements
	Budget execution control	There are corresponding control methods, the execution is related to the budget, no budget is not purchased, no budget is not spent
	Budget analysis and Assessment	Regularly analyze the variance rate of budget execution, establish a budget analysis and assessment system, and design corresponding assessment indexes
	Application of Budget Performance Evaluation Results	Follow up the completion of performance goals promptly, with reasonable reward and punishment measures
Purchase-side control	Bidding management system	The bidding management system is sound, the process is perfect, and there are corresponding control measures
	Supplier management	Two-way control of procurement and supplier collaboration, supervision of supplier qualification certificates
	Drugs, Materials and Medical Equipment Procurement Process and System C8	Establish and improve the procurement system and process of drugs, materials, and medical equipment, have corresponding control measures and separate incompatible positions from each other
Asset-side control	Asset dynamic supervision and inventory system	Dynamically grasp the storage and use of assets, and establish a regular inventory system
	Full tracking and management of high-value consumables	The whole process of approval and tracking management is implemented from application for access to use to realizing the write-off of doctor’s orders, and to support departments to provide feedback on the use of high-value consumables
	Medical equipment stand-alone accounting management	Realize one-machine-one accounting for key equipment, and evaluate equipment utilization efficiency
Expenditure business control	Expenditure approval management process and management system	Establish an expenditure approval process and system, which is related to the budget, no budget is not spent, and no budget is exceeded.
	Payment and acceptance consistency review	The payment business is associated with the storage document, one single code, to prevent repeated payment and wrong payment
	Check the authenticity of the bill	Validation and review of payment receipts
Contract management control	Contract review system	Establish a joint review system and process to jointly supervise the conclusion of contracts by multiple departments
	Centralized management of contracts	Set up an operation department to manage economic business contracts in a unified manner
	Contract performance supervision mechanism	Supervise the performance of the contract and ensure the consistency of contract execution and terms

The indicators are specifically designed for a wide array of public hospitals, ensuring their universal applicability, especially to tertiary hospitals at the prefecture and city level. However, due to the unique scope of indicators, special considerations are essential for some specialized hospitals. Nevertheless, the weighting method is universally applicable, suitable not only to various levels of public hospitals but also to specialized hospitals. This is attributable to the ability to employ the same method for weighting, despite the differing focal specialties of various hospitals.

### 3.2. Calculation results of subjective weights based on AHP

To establish the internal control index system for the public hospital supply chain, a panel of 10 experts in hospital supply chain internal control were invited to compare and score the 17 sub-criteria level indexes under the 5 categories. The purpose was to determine the standard layer and sub-criteria layer indexes. The relative importance between indexes was obtained, and the corresponding judgment matrix was constructed. The selection of the 10 experts was predicated on a set of predefined criteria, emphasizing their experience in hospital supply chain management, their academic pedigree, and their publication record within the domain of hospital supply chain management. Among these experts, four held doctoral degrees in supply chain management or related fields. Seven of them had dedicated over a decade to hospital supply chain management, and half were distinguished members of the China Association for Health Economics Management. The diversity and extensive expertise of this panel were instrumental in furnishing our research with in-depth and comprehensive insights, bolstering its robustness and credibility.

To effectively mitigate the subjective bias inherent in AHP, we adopted a multi-frequency expert scoring strategy. Specifically, we invited 10 experts to score multiple times and asked them to select the score they were most satisfied with from all their evaluations as the final score. Additionally, to further ensure the accuracy and consistency of the evaluations, we conducted a consistency test to ensure that all expert scores were within an acceptable range, thereby minimizing the impact of subjective bias to the greatest extent possible.

The evaluation opinions of 10 experts were integrated using the arithmetic mean and the AHP method was employed to analyze the internal control indexes of the public hospital supply chain. As a result, the subjective weight vector set ω_1_ was obtained, which is presented in Table 4. All data have passed the consistency test, ensuring the reliability of the evaluation process.

**Table 4:** Subjective weights of indexes.

Index	B1	B2	B3	B4	B5	Subjective weight
A	0.1762	0.3016	0.2236	0.1710	0.1276	
C_1_	0.2314					0.0408
C_2_	0.2076					0.0366
C_3_	0.3452					0.0608
C_4_	0.0927					0.0163
C_5_	0.1231					0.0217
C_6_		0.3024				0.0912
C_7_		0.1938				0.0585
C_8_		0.5038				0.1519
C_9_			0.4328			0.0968
C_10_			0.3796			0.0849
C_11_			0.1876			0.0419
C_12_				0.5917		0.1012
C_13_				0.2784		0.0476
C_14_				0.1299		0.0222
C_15_					0.3961	0.0505
C_16_					0.1716	0.0219
C_17_					0.4323	0.0552

### 3.3. Objective weight calculation results based on entropy weighting method

Six experts were invited to score the criteria and sub-criteria level indexes of the supply chain internal control. To ensure the objectivity and accuracy of the evaluation data, we adhered to the same selection criteria as before and chose an additional six experts who were not part of the original panel. Among these experts, two hold doctorate degrees in supply chain management or related fields. Five of them have dedicated over 10 years to hospital supply chain management, and all six are distinguished members of the China Health Economics Association.

The scoring results were used to construct an evaluation matrix (see Table 5 for details), from which the objective weight vector set ω2 of each index was obtained using the entropy weighting method calculation formula. The obtained objective weight vector set ω_2_ is shown in Table 6.

**Table 5:** Expert scoring results.

Expert	**C**_**1**_	**C**_**2**_	**C**_**3**_	**C**_**4**_	**C**_**5**_	**C**_**6**_	**C**_**7**_	**C**_**8**_	**C**_**9**_	**C**_**10**_	**C**_**11**_	**C**_**12**_	**C**_**13**_	**C**_**14**_	**C**_**15**_	**C**_**16**_	**C**_**17**_
1	8	7	9	5	6	8	6	10	9	7	5	8	7	6	6	5	7
2	8	7	8	5	6	7	5	8	8	7	5	8	7	5	6	5	7
3	6	6	7	5	5	6	6	6	7	7	6	6	6	6	6	5	6
4	6	7	5	5	6	6	6	5	7	6	5	7	6	6	6	4	7
5	7	6	7	4	6	8	6	7	8	6	5	6	7	6	5	5	6
6	9	5	6	5	5	7	5	9	6	8	5	7	6	5	5	5	6

**Table 6:** Objective weights of design indexes.

Index	Information entropy	Degree of difference	Weights
C_1_	0.99363699	0.00636301	0.096815241
C_2_	0.996019759	0.003980241	0.060560637
C_3_	0.990394973	0.009605027	0.146143568
C_4_	0.998256948	0.001743052	0.026521103
C_5_	0.998027399	0.001972601	0.030013757
C_6_	0.996190309	0.003809691	0.057965676
C_7_	0.998027399	0.001972601	0.030013757
C_8_	0.985304501	0.014695499	0.223596725
C_9_	0.995426608	0.004573392	0.069585628
C_10_	0.99718981	0.00281019	0.042757948
C_11_	0.998605717	0.001394283	0.021214469
C_12_	0.996190309	0.003809691	0.057965676
C_13_	0.998347153	0.001652847	0.025148599
C_14_	0.998027399	0.001972601	0.030013757
C_15_	0.998027399	0.001972601	0.030013757
C_16_	0.998256948	0.001743052	0.026521103
C_17_	0.998347153	0.001652847	0.025148599

### 3.4. Comprehensive weight calculation results based on game theory combination weighting method

By combining the subjective and objective weight calculation results and applying the relevant formula of the game theory combination weighting method, the comprehensive weight of each index was calculated using the game combination weighting method. The comprehensive weight of the criterion layer indexes was further calculated to construct the reference standard of the evaluation plan, which is presented in Table 7.

**Table 7:** Composite weighting results.

Index	Analytic hierarchy process results	Entropy weighting method results	Game theory Combination weighting method results
C_1_	0.0408	0.09681524	0.096668
C_2_	0.0366	0.06056064	0.060498
C_3_	0.0608	0.14614357	0.145919
C_4_	0.0163	0.0265211	0.026494
C_5_	0.0217	0.03001376	0.029992
C_6_	0.0912	0.05796568	0.058053
C_7_	0.0585	0.03001376	0.030089
C_8_	0.1519	0.22359673	0.223408
C_9_	0.0968	0.06958563	0.069657
C_10_	0.0849	0.04275795	0.042869
C_11_	0.0419	0.02121447	0.021269
C_12_	0.1012	0.05796568	0.058079
C_13_	0.0476	0.0251486	0.025208
C_14_	0.0222	0.03001376	0.029993
C_15_	0.0505	0.03001376	0.030068
C_16_	0.0219	0.0265211	0.026509
C_17_	0.0552	0.0251486	0.025228

Leveraging the previously derived weight results, this study conceived a robust scheme for managing internal control within public hospital supply chains. This scheme was brought to life at the Weifang Maternal and Child Health Hospital, serving as a practical testament to the proposed concept. The effectiveness of this real-world application was then scrutinized through a fuzzy evaluation method, providing a comprehensive assessment of its performance. What follows is a detailed exposition of the modifications made to the scheme and the outcomes gathered from its practical deployment.

### 3.5. Scheme description and implementation details

Weifang Maternal and Child Health Hospital (referred to as F Hospital) is a specialized public hospital of tertiary first-class level with a development history of over 60 years. Guided by the weightings of various indicators provided by the combination weighting method based on game theory, we have implemented the following improvements to the internal control management of the hospital’s supply chain:

To enhance control on the planning side, we introduced a centralized management system for all functional departments. We concurrently adopted an adaptive management system that supports a variety of procurement strategies, tailored to meet the needs of diverse stakeholders. This innovative system ensures that procurement processes closely align with the real-time demands of our operations. To enhance control on the procurement side, we instituted a comprehensive supplier management protocol. This new system meticulously governs the process of supplier selection, evaluation, and performance management, ensuring consistency and high standards throughout the supply chain. We made substantial use of a Hospital Resource Planning (HRP) system to link bidding procedures with our established budgets, which fostered a unique synergy between these two previously independent processes. This integration allowed us to achieve an unprecedented level of proactive control over the bidding process. Furthermore, we introduced an early warning system for inventory control, notifying key personnel of both minimum and maximum stock thresholds. This proactive approach not only ensures smooth operations but also promotes economic efficiency by maintaining optimal inventory levels. Regarding the control on the asset side, we employed the logistics and fixed assets modules of the HRP system, leading to the establishment of secondary warehouses in clinical departments. This system transformation enabled the hospital to enact effective chargeable materials write-off policies, manage non-chargeable materials quotas, and maintain comprehensive traceability management for high-value consumables. As for contract management, we streamlined the process by placing hospital procurement contracts under the centralized control of the operations management department. The contract counter-signing process was seamlessly integrated into the HRP system. This approach empowered different departments, such as bidding, auditing, finance, discipline inspection, and legal affairs, to effectively perform their roles in the counter-signing process. Moreover, it ensured complete transparency and accessibility of every phase of contract execution within the system.

### 3.6. Comprehensive evaluation based on fuzzy evaluation method

As of now, the scheme outlined above has been in a state of continuous refinement and implementation for close to 4 years, spanning from September 2019 to April 2023. In this research, we have made use of fuzzy evaluation techniques to perform a holistic comparison. This includes a detailed appraisal of the internal supply chain control system within Hospital F as it was in 2019 and its subsequent transformation following the implementation of our proposed scheme in 2023. The specifics of this evaluation process are elaborated in the following sections.

#### 3.6.1. Comprehensive evaluation of supply chain internal control in F hospital in 2019

First, determine the comment set *V* = {very satisfied, satisfied, average, dissatisfied, very dissatisfied}for the internal control scheme of the supply chain and assign it *V* = {100, 80, 60, 40, 20}.

Second, determine the comprehensive weight vector of indexes at different levels (18). According to the comprehensive weights of the indexes of the game combination weighting above, the comprehensive weight vector of the criterion layer is *W_A_* = (0.096668, 0.060498, 0.145919, 0.026494, 0.029992, 0.058053, 0.030089, 0.223408, 0.069657, 0.042869, 0.021269, 0.058079, 0.025208, 0.029993, 0.030068, 0.026509, 0.025228).

Third, to construct the fuzzy comprehensive evaluation matrix of the sub-criteria level of the internal control index of the F Hospital supply chain, we invited the original 10 experts who evaluated the indicators to assess and score the supply chain internal control construction of F Hospital. The experts determined the relevance of evaluation comments based on their frequency. The matrices N1 to N5 represent the comprehensive evaluations for plan-side control, purchase-side control, asset-side control, expenditure business control, and contract management control.


$$\begin{array}{c} \;N_{1}=\left(\begin{array}{ccccc} 0.6 & 0.3 & 0.1 & 0 & 0\\ 0.5 & 0.4 & 0.1 & 0 & 0\\ 0.3 & 0.4 & 0.3 & 0 & 0\\ 0.2 & 0.4 & 0.4 & 0 & 0\\ 0 & 0.4 & 0.4 & 0.2 & 0 \end{array}\right) \end{array}$$

$$\begin{array}{c} \;N_{2}=\left(\begin{array}{ccccc} 0.3 & 0.5 & 0.2 & 0 & 0\\ 0 & 0.5 & 0.4 & 0.1 & 0\\ 0.6 & 0.4 & 0 & 0 & 0 \end{array}\right) \end{array}$$

$$\begin{array}{c} \;N_{3}=\left(\begin{array}{ccccc} 0.4 & 0.5 & 0.1 & 0 & 0\\ 0.8 & 0.2 & 0 & 0 & 0\\ 0.2 & 0.6 & 0.2 & 0 & 0 \end{array}\right) \end{array}$$

$$\begin{array}{c} \;N_{4}=\left(\begin{array}{ccccc} 0.9 & 0.1 & 0 & 0 & 0\\ 0.6 & 0.3 & 0.1 & 0 & 0\\ 0.1 & 0.6 & 0.3 & 0 & 0 \end{array}\right) \end{array}$$

$$\begin{array}{c} \;N_{5}=\left(\begin{array}{ccccc} 0.5 & 0.5 & 0 & 0 & 0\\ 0.3 & 0.6 & 0.1 & 0 & 0\\ 0 & 0.5 & 0.4 & 0.1 & 0 \end{array}\right) \end{array}$$

Based on this, the total score of F Hospital supply chain internal control construction can be obtained:


$$\begin{array}{c} \;P=HV=85.69 \end{array}$$

#### 3.6.2. Comprehensive evaluation of supply chain internal control in F hospital in 2023

Building upon the assessment conducted in 2019, a subsequent evaluation of the supply chain internal control system was undertaken at F Hospital in April 2023. The evaluation aimed to measure the efficacy and continued improvement of the internal control scheme over a span of 4 years, as well as to identify areas that could be further optimized. A holistic comparison, employing the same fuzzy evaluation techniques, was utilized to accurately gage the evolution of the internal control system.

Similar to the 2019 evaluation, an expert panel of 10 supply chain internal control specialists was convened to conduct the evaluation and score the supply chain internal control construction of F Hospital. Subsequently, the fuzzy comprehensive evaluation matrix for each criterion level was generated (N_1_-N_5_).


$$\begin{array}{c} \;N_{1}=\left(\begin{array}{ccccc} 0.8 & 0.2 & 0 & 0 & 0\\ 0.7 & 0.3 & 0 & 0 & 0\\ 0.6 & 0.3 & 0.1 & 0 & 0\\ 0.6 & 0.3 & 0.1 & 0 & 0\\ 0.1 & 0.6 & 0.2 & 0.1 & 0 \end{array}\right) \end{array}$$

$$\begin{array}{c} \;N_{2}=\left(\begin{array}{ccccc} 0.5 & 0.4 & 0.1 & 0 & 0\\ 0.2 & 0.6 & 0.2 & 0 & 0\\ 0.7 & 0.3 & 0 & 0 & 0 \end{array}\right) \end{array}$$

$$\begin{array}{c} \;N_{3}=\left(\begin{array}{ccccc} 0.6 & 0.3 & 0.1 & 0 & 0\\ 0.8 & 0.2 & 0 & 0 & 0\\ 0.3 & 0.6 & 0.1 & 0 & 0 \end{array}\right) \end{array}$$

$$\begin{array}{c} \;N_{4}=\left(\begin{array}{ccccc} 0.9 & 0.1 & 0 & 0 & 0\\ 0.7 & 0.2 & 0.1 & 0 & 0\\ 0.2 & 0.6 & 0.2 & 0 & 0 \end{array}\right) \end{array}$$

$$\begin{array}{c} \;N_{5}=\left(\begin{array}{ccccc} 0.7 & 0.3 & 0 & 0 & 0\\ 0.5 & 0.4 & 0.1 & 0 & 0\\ 0.1 & 0.6 & 0.2 & 0.1 & 0 \end{array}\right) \end{array}$$

Based on the resultant evaluation matrices, the total score of F Hospital’s supply chain internal control construction for 2023 was computed and is given by:


P=HV=90.75.

This score, higher than the previous score of 85.69 obtained in 2019, underscores the successful refinement and implementation of the internal control scheme. The assessment showed an increased satisfaction level across all criteria, signifying the effectiveness of the continuous improvement approach employed in F Hospital’s supply chain internal control system. It is important to note, however, that this system is not static, and continued refinement will further enhance its effectiveness in the coming years.

## 4. Discussions

After evaluations in 2019 and 2023, a significant enhancement was observed in the internal control system of F Hospital’s supply chain, evidenced by the score increase from 85.69 to 90.75. This change is not merely a superficial increase in numbers; it reflects profound real-world implications. Firstly, this growth signifies that during this period, we successfully optimized key processes and operations, thereby enhancing efficiency and output. Secondly, the rise in scores also indicates a notable increase in the satisfaction levels of both employees and patients, suggesting that our service quality has been acknowledged and appreciated. Most importantly, this consistent positive trend offers us a clear direction, validating that our investments in research and development are on the right track, laying a solid foundation for future strategic planning and decision-making.

During the pre-implementation and post-implementation phases of the scheme, we noted that core elements like the hospital’s infrastructure, staff size, market trends, policy adjustments, technological advancements, and supplier relationships remained relatively stable over the years. This indicates that the increase in scores was not due to changes in other external conditional variables. Importantly, areas directly associated with the scheme witnessed a pronounced score increment, while those not emphasized in the scheme experienced only modest growth. Based on these observations, we have substantial grounds to believe that the notable score increase is intrinsically linked to the refinements made according to the proposed scheme. The score growth over these 4 years not only validates the accuracy of the metrics used in formulating the scheme but also accentuates the efficacy of the internal control design we instituted. The significant progress made within this period provides compelling evidence for the practicality and feasibility of the game theory combination weighting method in the realm of hospital supply chain internal control. This approach not only offers a balanced and precise assessment of the internal control system but also paves the way for the development of a more refined and scientifically rigorous control scheme.

In conclusion, the application of the game theory combination weighting method in designing hospital supply chain internal control systems has been empirically validated in F Hospital. This approach not only fortifies the scientific rigor of the control scheme design but also guarantees its sustained optimization and effectiveness. However, it’s imperative to note that the findings of this study, being based solely on data from a single hospital, might have inherent limitations in terms of generalizability and applicability across diverse hospital settings. This suggests that the proposed method may require further validation and adjustments in different hospital contexts. Despite these potential constraints, the method can still be regarded as an innovative and reliable tool for the design of internal control mechanisms within public hospital supply chains.

## 5. Conclusion

This study presents a sophisticated approach to the enhancement of public hospital supply chain management by developing an internal control index system grounded in the game theory combination weighting method. This novel method addresses the issue of index duplication and augments the comprehensiveness and accuracy of the system. By transcending the constraints of traditional top-down designs, it encourages a more scientific, methodical approach to managing the supply chain internal control. This study utilizes Hospital F as a practical subject, employing the Fuzzy Evaluation Method to corroborate the feasibility of applying the Game Theory Combination Weighting Method in the context of hospital supply chain internal control. By evaluating the hospital before and after the implementation of the proposed design scheme, the effectiveness of the combined approach can be ascertained. The insights gleaned from this analysis can furnish valuable methodologies and pathways for future enhancements of internal control within hospital supply chains. Moreover, these findings can contribute to the broader advancement of supply chain management within the healthcare industry.

Despite the meaningful strides made in this research, we acknowledge its limitations and the need for further investigation. Firstly, the intricacy of supply chains and internal control systems warrants additional exploration to ascertain the comprehensiveness of the proposed internal control index system. Secondly, while we employed the Entropy Weighting Method to mitigate the influence of subjective biases in the Analytic Hierarchy Process, the weighting and evaluation results are still susceptible to expert preferences. This inherent bias is inevitable and must be considered. Thirdly, our findings are derived from a singular case study, which may not encompass the diversity and intricacy of various hospital settings. Validation across multiple cases is essential to bolster the generalizability of our results. It should be emphasized that the proposed method and indicators for internal control within public hospital supply chains were designed with a broad perspective, aiming to cater to the general needs of public hospitals. However, individual hospitals, due to variations in their economic conditions and socio-cultural environments, may face unique challenges and requirements. For instance, specialty hospitals should consider their target audience and place emphasis on certain indicators accordingly. Therefore, while the foundational principles of the method are designed to be universally applicable, some customization and adjustments might be necessary based on the specific context and needs of each public hospital.

The future work will aim to address these limitations and further develop and refine the internal control index system. This study is an initial step toward a more comprehensive and scientifically robust evaluation of internal control systems within the hospital supply chain. The insights and outcomes from this research contribute to the ongoing discourse in this field and lay a foundation for future advancements.

## Data availability statement

The original contributions presented in the study are included in the article/supplementary material, further inquiries can be directed to the corresponding author.

## Ethics statement

This research was conducted following the ethical standards of the scientific community. All the data were collected with proper permissions, respecting confidentiality and privacy. Expert interviews were carried out with informed consent, and all practices respected the autonomy and rights of the participants. The study received appropriate ethical approval from relevant institutional bodies.

## Author contributions

ZY conceptualized and designed the study, conducted the literature review, collected and analyzed the data, and wrote the manuscript and is accountable for all aspects of the work and ensures that questions related to the accuracy or integrity of any part of the work are appropriately investigated and resolved.
